# Sensitivity of anti-filarial antibodies for lymphatic filariasis surveillance: Insights from a serological survey in Samoa in 2018

**DOI:** 10.1371/journal.pntd.0012835

**Published:** 2025-01-30

**Authors:** Harriet L. S. Lawford, Benn Sartorius, Helen J. Mayfield, Filipina Amosa-Lei Sam, Satupaitea Viali, Tito Kamu, Robert Thomsen, Colleen L. Lau

**Affiliations:** 1 UQ Centre for Clinical Research, Faculty of Health, Medicine, and Behavioural Sciences, The University of Queensland, Brisbane, Queensland, Australia; 2 National University of Samoa, Apia, Samoa; 3 Oceania University of Medicine, Apia, Samoa; 4 Tupua Tamasese Meaole Hospital, Apia, Samoa; 5 Ministry of Health, Apia, Samoa; TOBB Economics and Technology University Faculty of Medicine: TOBB Ekonomi ve Teknoloji Universitesi Tip Fakultesi, TÜRKIYE

## Abstract

**Background:**

Sensitive diagnostic tools that signal lymphatic filariasis (LF) transmission are needed to monitor the progress of LF elimination programs. Anti-filarial antibody (Ab) markers could be more sensitive than antigen (Ag) point-of-care tests for monitoring LF transmission in some settings. This study aimed to investigate the sensitivity of anti-filarial Abs for detecting signals of LF transmission in Samoa by i) investigating the sensitivity and specificity of Ab to identify Ag-positives; ii) estimating the average number needed to test (NNTest^av^) to identify LF-seropositives (seropositive for Ag and/or any Ab), and iii) compare the efficiency of the different serological indicators by target age group and sampling design.

**Methods:**

A community-based serological survey of participants aged ≥5 years was conducted 1.5-3.5 months following the first round of triple-drug mass drug administration in Samoa in 2018, covering 35 primary sampling units (PSUs) (30 randomly selected and five purposively selected ‘suspected hotspots’). Ag-positivity was detected using Alere Filariasis Test Strips, and Ab-seropositivity (*Bm14*, *Wb123*, *Bm33* Abs) were measured using multiplex bead assays. Seroprevalence was adjusted for study design and standardised for age and gender. NNTest^av^ was calculated using the formula 1/p, where p was the adjusted seroprevalence for each subgroup.

**Results:**

Of 3795 participants (mean age: 20.7; 51.2% female), 1892 (49.9%) were LF-seropositive. If Ag alone was used to identify LF-seropositives, only 5% (117/1892) would be identified. Of the three Ab seromarkers, *Bm14* Ab had the highest area under the Receiver-Operating Characteristic Curve ([ROC]=0.88) to classify participants as Ag-positive, followed by *Wb123* Ab (ROC=0.83) and *Bm33* Ab (ROC=0.76). Participants aged ≥10 years had lower NNTest^av^ compared to participants aged 5-9 years for all seromarkers. NNTest^av^ was lower in purposively versus randomly selected PSUs.

**Conclusions:**

All Ab seromarkers had high ROC values to classify patients as Ag-positive and may be useful tools for LF surveillance in some settings. However, further research is required to fully understand how best Ab serosurveillance can be incorporated into LF elimination programmes.

## Author summary

The Global Program to Eliminate Lymphatic Filariasis (GPELF) is one of the largest global programs for neglected tropical disease elimination and since its formation, the number of lymphatic filariasis (LF) infections has been reduced by 74% globally. However, several countries including Samoa have reported persistent foci of LF transmission. For LF elimination to succeed, detecting and treating infected individuals is critical. Whilst point-of-care tests to detect filarial antigen (Ag) are recommended for use in LF elimination programs, there are concerns about their reduced sensitivity in some settings. Recent evidence suggests that anti-filarial antibody (Ab) markers could be more sensitive measures of transmission. In this study, we found that only 3.7% (n=117) of participants were Ag-positive, whilst 57.8% (n=1889) of participants tested positive to at least one Ab, suggesting a significant proportion of LF-seropositive participants would be missed if Ag testing alone was used. Of the three Ab seromarkers, *Bm14* Ab had the highest area under the Receiver-Operating Characteristic Curve (0.88) to classify participants as Ag-positive. Furthermore, in this study we found that the average number needed to test (NNTest^av^) to identify one positive participant for all seromarkers was lower for all Abs compared to Ag, decreased as age increased, and was lower in suspected hotspots. Serosurveillance with Abs could provide a valuable additional monitoring tool for countries aiming for elimination. Our NNTest^av^ results can help inform guidelines to target limited resources towards conducting surveillance among older individuals in suspected hotspots, thereby increasing the efficiency of surveys to identify residual foci of LF transmission.

## Introduction

Lymphatic filariasis (LF) is a neglected tropical disease (NTD) transmitted by mosquitoes and caused by the roundworms *Brugia malayi*, *Brugia timori*, and *Wuchereria bancrofti*. Microfilariae (Mf), transmitted via the bite of an infected mosquito, go on to develop into adult worms that nest in the lymphatic vessels and can lead to impairment of the lymphatic system [1]. If left untreated, infection can result in severe lymphedema (and, in males, scrotal hydrocele) leading to permanent disability and disfigurement [2]. As of 2021, an estimated 882.5 million people in 44 countries were at risk of LF, with *W. bancrofti* responsible for 90% of cases [2].

Mass drug administration (MDA) is the World Health Organization’s (WHO) recommended preventive chemotherapy strategy for eliminating LF. MDA regimens reduce the Mf density in the bloodstream and the intensity of infection in communities to a level where transmission is unlikely to be sustained [2,3]. WHO currently recommends the use of point-of-care tests to detect circulating *W. bancrofti* filarial antigen (Ag) produced by adult worms as the standard serological indicator for LF programmatic surveys [4]. However, there are concerns that Ag testing may have lower sensitivity to identify infections when Ag prevalence is very low, e.g., following effective MDA. This can potentially lead to failure to identify signals of transmission [5–7], particularly among subgroups that are known to have lower Ag prevalence, e.g., young children [8].

Serological assays that test for immunoglobulin G (IgG) antibodies (Ab) to pathogen-specific Ag can provide evidence of an individual’s infection or vaccination status [9]. In general, IgM Ab appear in the early period of an infection and are considered indicative of current/recent infection, whilst IgG Ab usually develop a week or more post-infection and can persist for long periods, sometimes for life [10]. Thus, IgG Ab responses are generally considered markers of middle/late-stage active infection, past infection, or immunity [9,10].

Serological tests that detect the presence of anti-filarial IgG Abs (including *Wb123* Ab, *Bm14* Ab, and *Bm33* Ab) have been found to be more sensitive for detecting LF infection than Ag testing alone [5,6]. Studies have shown that Ab responses may develop before patent infection or antigenemia [11], thus Ab testing may provide an earlier indication of infection or identify asymptomatic individuals. Thus, at the population level, seroprevalence of anti-filarial Abs may provide earlier signals of LF transmission and/or resurgence and enable a timelier response compared to using Ag prevalence alone [5,12]. Further, *Wb123* is a larval Ag, meaning that *Wb123* Ab-positive results are more likely to be associated with exposure to infected mosquitoes (and therefore an indicator of ongoing transmission) than *Bm14* Ab-positive or *Bm33* Ab-positive results [13]. A study from American Samoa found that the average Number Needed to Test (NNTest^av^) to identify one LF-seropositive individual was lower for Abs than for Ag, reinforcing the potential value of LF Ab serosurveillance [14].

However, the serological profile for LF is complex because of the long lifespan of adult filarial worms; individuals may be infectious (microfilaraemic) for many years, and Abs, Ag, and Mf could be detected concurrently, including in asymptomatic infections. On the other hand, an individual may still be seropositive for Ag and Abs for months to years following a cleared infection [15]. Currently, there are knowledge gaps related to the sensitivity and specificity of Abs to identify active LF infection, and whether Abs can be used to discriminate between active, recently cleared, or past LF infection.

Samoa is a tropical island nation in the Western Pacific Region with a population of 222,382 in 2022 [16]. Despite substantial reductions in LF transmission following multiple national and targeted MDA rounds, most recently in September 2023, Samoa remains endemic for LF [17,18]. A nationwide Transmission Assessment Survey (TAS) was conducted in 2013, in which two of three evaluation units (EU) passed target thresholds, and a second TAS took place in 2017 where all three EUs failed [18]. In 2017, the WHO released guidelines on *‘Alternative MDA Regimens to Eliminate LF’* following several countries reporting suboptimal results after MDA with standard two-drug regimens [19]. In countries endemic for LF but without onchocerciasis or loa loa that were using diethylcarbamazine and albendazole (DA) for MDA, recommendations were to switch to annual triple-drug MDA using ivermectin, diethylcarbamazine, and albendazole (IDA) in settings with (i) implementation units that had not started, or had fewer than four effective DA rounds; (ii) implementation units that had not met epidemiological thresholds in sentinel and spot-check site surveys or in TAS despite meeting drug coverage target; and (iii) communities where post-MDA or post-validation surveillance identified infection suggesting local transmission [19]. In 2018, Samoa was the first country in the world to distribute IDA nationwide.

The operational research project Surveillance and Monitoring to Eliminate LF and Scabies from Samoa (SaMELFS) was established to monitor the impact of nationwide triple-drug MDA on LF transmission in Samoa [18]. The first SaMELFS survey took place in 2018, 1.5-3.5 months following the first national distribution of IDA, and was considered a baseline survey for measuring the impact of triple-drug MDA on LF prevalence. A previously published paper reported Ag prevalence at the population level, as well as by age group (5-9 years vs ≥10 years), and found an adjusted overall Ag prevalence of 4.0% (95% confidence interval [CI]: 3.1-7.0), and a three-fold higher Ag prevalence among participants aged ≥10 years compared to those aged 5-9 years [18].

This study complements our previous paper that reports baseline Ab seroprevalence (*Bm14*, *Wb123*, and *Bm33* Abs) estimates from the SaMELFS 2018 survey [20]. The current study aimed to investigate the potential utility of anti-filarial Abs to guide and support decision-making for national LF elimination programs. Our objectives were to i) investigate the sensitivity and specificity of individual Abs and combinations of Abs to identify Ag-positives; and ii) estimate the average number needed to test (NNTest^av^) to identify LF-seropositives (seropositive for Ag and/or Ab) and iii) compare the efficiency of the different serological indicators by target age group and sampling design.

## Methods

### Ethics statement

Ethics approvals were granted by the Samoa Ministry of Health and The Australian National University Human Research Ethics Committee (protocol 2018/341) and ratified by The University of Queensland Human Research Ethics Committee. The study was conducted in close collaboration with the Samoa Ministry of Health, the WHO country office in Samoa, and the Samoa Red Cross. Written informed consent was obtained from adult participants. For participants aged <18 years, verbal assent was obtained from the child, and formal written consent was obtained from a parent or guardian.

### Data source

Samples were collected during the baseline SaMELFS survey in 2018. The SaMELFS study design has been described in detail [18]. Briefly, participants aged ≥5 years were eligible for inclusion in a community-based serosurvey that was conducted in 35 primary sampling units (PSUs), 30 of which were randomly selected and five were purposively selected by the Ministry of Health as suspected hotspots based on historical surveys. A convenience survey of children aged 5-9 years was also conducted in each PSU. Demographic information was collected from each participant using a standardised electronic questionnaire and a finger prick blood sample of up to 400 μL was collected and used to test for Ag using Alere Filariasis Test Strips (FTS) (Abbott, Scarborough, ME) [21] and to prepare dried blood spots (DBS) for multiplex bead assays (MBA). For Ag-positive samples, thick blood smears were prepared for microscopic examination for Mf.

### Multiplex bead assay

DBS were eluted into 96-well plates and then diluted to a final concentration of 1:400 [22]. All samples were tested using MBA for anti-filarial Abs *Bm14*, *Bm33*, and *Wb123* [23]. Sample plates were read on a Bio-Plex 200 instrument (Bio-Rad, Hercules, CA). For internal quality control purposes, four controls were used for the MBA analyses: a buffer blank containing the assay buffer only (to subtract any background noise), two pools of reference sera from known Ab-positives, and finally a negative control serum with known negative LF status. For each antigen, the mean plus three standard deviations of the median fluorescence intensity-background (MFI-bg) of a panel of 81 individuals from non-endemic regions were used to determine a threshold for seropositivity.

### Definition of subgroups used in the analysis

Participants were considered Ag-positive if they had a positive FTS result. Participants were considered Ab-positive if they were seropositive to at least one of three Abs tested. Participants were considered LF-seropositive if they were seropositive to Ag and/or any Ab.

### Statistical analysis

Data were analysed using Stata statistical software (StataCorp, Version 17.0, College Station, TX). Adjustment for selection probability and clustering was based on the 2016 Samoa Census [24] and performed using the ‘svyset’ command in Stata with PSU as the unit of clustering. Age group and gender standardized weights were applied using information from the 2016 Samoa Census [24]. Prevalence estimates for the two main age groups were adjusted for selection probability and clustering and standardized for gender but not age. The baseline seroprevalence estimates for all ages ≥5 years were adjusted for selection probability and clustering and standardized for gender (except when calculating gender-specific prevalence) and age using 5-year age bands. Further detail on standardization and adjustments, including the values used, have been described previously [13].

Correlation between the adjusted seroprevalence of Ag, *Bm14* Ab, *Wb123* Ab, and *Bm33* Ab at the PSU level among all participants, and by ages 5-9 years and ≥10 years were investigated using Spearman’s correlation coefficient (Stata command ‘pwcorr’). Using Ag as the reference, the sensitivity and specificity of individual Abs and combinations of Abs were assessed using a diagnostic test module (‘diagt’) in Stata. A multinomial logistic regression model was used to investigate associations between predictor variables (age and PSU selection [randomly or purposively selected]), and the outcome (single and combination of Ab and/or Ag seropositivity). The results of the multinomial logistic regression were expressed as relative risk ratio (RRR) for each variable, with 95% CI and *p*-values calculated with an alpha level of 0.05. The ratio of Ag-positive to Ab-positive participants were calculated using the ‘ratio’ command in Stata incorporating sampling weights and bootstrapped confidence intervals based on 1000 replications. We chose bootstrapped confidence intervals given the non-random sampling design for the purpose component.

NNTest measures are easier to explain than other measures of effect and may be more easily interpreted by programmatic decision-makers [14]. The NNTest^av^ to identify one LF-seropositive (with various degrees of probability) was calculated using the formula 1/p, where p was the adjusted seroprevalence for Ag and each Ab. The number needed to test to provide a 50% chance of identifying at least one positive result (NNTest^50^), and a 95% chance of identifying at least one positive result (NNTest^95^) were calculated using formulae described elsewhere [14]. Of note, NNTest^50^ and NNTest^95^ are used here to provide examples of the sample size required for different degrees of certainty (50% and 95% certain) that positive cases are not missed if they are present.

## Results

Overall, 3940 participants aged ≥5 years had DBS collected for MBA. Of these, Ab results were not available for 89 (2.3%) participants: 35 samples had a low bead count (one of which also had an invalid FTS result), no reason was given for 21 samples (of which 14 had no FTS result recorded and 7 were Ag-negative), no consent was given for 17 samples, and 16 duplicate samples were excluded (of which three were Ag-positive and Mf-positive, and two were Ag-positive and Mf-negative). A further 56 participants without an FTS result were excluded (30 had invalid FTS results, 16 did not have enough blood, and 10 had no reason given), giving a total sample of 3795 participants included in the analyses (S1 Fig).

### Study population demographics and adjusted Ag and Ab prevalence

The demographics of the study population and the prevalence of Ag and Ab in the sample has been described elsewhere [25]. In brief, the mean age of participants was 20.7 years (range: 5-90 years) and 51.2% were female. Fourteen percent of participants were recruited from the five purposively selected PSUs, with the remaining participants recruited from the 30 randomly selected PSUs. There were no significant differences in demographic characteristics between purposively selected and randomly selected PSUs (S1 Table). However, a significantly higher proportion from purposively selected PSUs had taken MDA in the past (80.7% vs 62.6%; p < 0.001) and had spent their whole life in Samoa (95.0% vs 88.3%; p < 0.001). In total, 117 (3.7%) of participants were Ag-positive and 1892 (57.9%) were LF-seropositive. Refer to S2 Table for the adjusted seroprevalence for Ag and Ab seromarkers.

### Correlation between Ag and Ab seroprevalence by age group at the PSU level

Significant association and moderate correlation were seen between Ag prevalence and Ab prevalence at the PSU level (*Bm14* Ab: *rho*=0.754; *Wb123* Ab: *rho*=0.660; *Bm33* Ab: *rho*=0.613), with the strongest correlation seen between *Bm14* Ab prevalence and Ag prevalence. When restricted to participants aged 5-9 years, the association remained significant, but correlation was weaker (*Bm14* Ab: *rho*=0.470; *Wb123* Ab: *rho*=0.579; *Bm33* Ab: *rho*=0.589). Correlation was greatest when restricted to participants aged ≥10 years (*Bm14* Ab: *rho*=0.806; *Wb123* Ab: *rho*=0.723; *Bm33* Ab: *rho*=0.655) and strongest between *Bm14* Ab and Ag prevalence (S2 Fig).

At the PSU level, the strongest correlation between Abs was seen between *Bm14* Ab and *Wb123* Ab, with *rho*=0.912 in all participants aged ≥5 years, and *rho*=0.935 in participants ≥10 years old. For participants aged 5-9 years, the strongest correlation was between *Wb123* Ab and *Bm33* Ab (*rho*=0.782) (S3 Fig).

### Potentially ‘missed’ LF-seropositives

Of the 1892 LF-seropositive participants, only 117 (5.9%) were Ag-positive; nearly 95% of LF-seropositives would be ‘missed’ if tested for Ag alone. Overall, 1853 (98.1%) participants tested positive to *Bm33* Ab and/or *Wb123* Ab, thus the fewest LF-seropositives would be ‘missed’ (1.9%) if tested for these seromarkers. Additional calculations are presented in S3 Table.

### Seropositivity profile of participants testing negative to different Ag and Ab combinations

**Fig 1:**
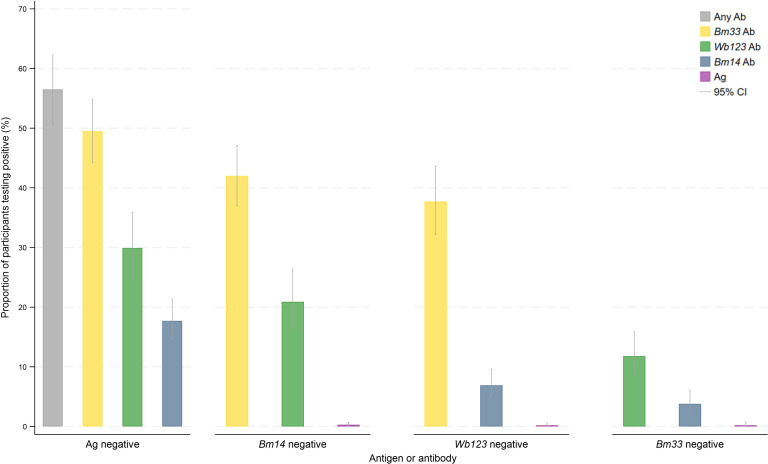
Proportion of participants testing positive for antigen (Ag) and individual antibodies (Abs) if the participants tested negative for (i) Ag, (ii) *Bm14* Ab, (iii) *Wb123* Ab, or (iv) *Bm33* Ab, adjusted for survey design and standardised by age and sex, Samoa 2018.

**Fig 2:**
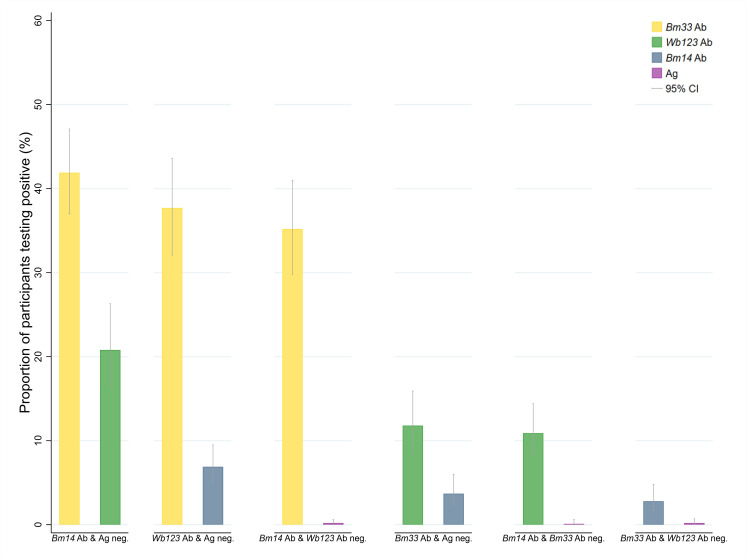
Proportion of participants testing positive for combinations of antigen (Ag) and antibodies (Abs) if the participants tested negative for (i) *Bm14* Ab and Ag, (ii) *Wb123* Ab and Ag, (iii) *Bm14* Ab and *Wb123* Ab, (iv) *Bm33* Ab and Ag, (v) *Bm14* Ab and *Bm33* Ab, or (vi) *Bm33* Ab and *Wb123* Ab, adjusted for survey design and standardised by and sex, Samoa 2018.

Fig 1 and S4 Table show the seropositivity profile of individuals who tested negative to each seromarker, adjusted for survey design and standardised by age and sex. Fifty-seven percent of Ag-negative participants were positive for at least one Ab, whilst only 0.3%, 0.2%, and 0.2% of *Bm14* Ab-negative, *Wb123* Ab-negative, and *Bm33* Ab-negative participants, respectively, were Ag-positive. Fig 2 and S4 Table display the proportion of participants testing positive for different combinations of Ag and Abs, adjusted for survey design and standardised by age and sex.

### Sensitivity and specificity of Abs for detecting Ag-positives

**Fig 3:**
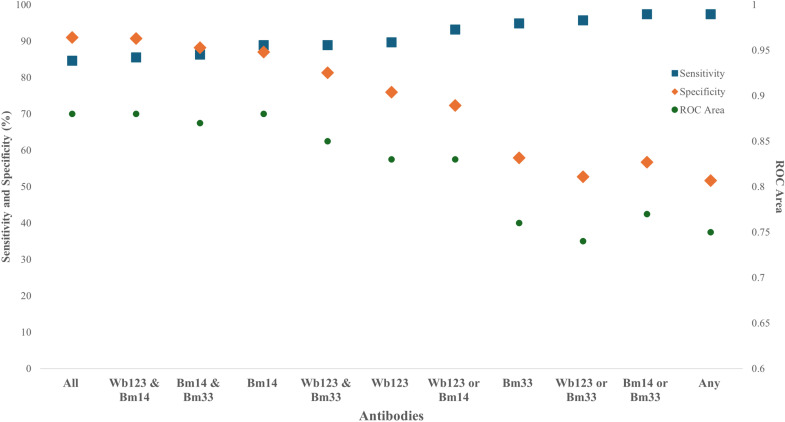
Sensitivity, specificity, and area under the receiver-operating characteristic curve (ROC) of individual antibodies (Abs), and combinations of Abs, to classify participants as antigen-positive, Samoa 2018. ROC = area under the receiver-operating characteristic curve.

Table 1 and Fig 3 shows the sensitivity, specificity, positive predictive value (PPV), negative predictive value (NPV), and area under the Receiver-Operating Characteristic Curve (ROC) for Ag-positivity based on individual Ab-positivity as well as positivity for different combinations of Abs. Of the three Ab seromarkers, *Bm14* Ab had the highest ROC (ROC=0.88) to classify participants as Ag-positive, followed by *Wb123* Ab (ROC=0.83) and *Bm33* Ab (ROC=0.76). When combinations of seromarkers were considered, testing positive to both *Bm14* Ab and *Wb123* Ab, or to all three Abs, had the highest ROC (0.88). Comparisons of the sensitivity, specificity, PPV, NPV, and ROC by age group (5-9-year-olds and ≥10-year-olds) are presented in S5 Table and S4 Fig.

**Table 1:** Sensitivity, specificity, positive predictive value (PPV), negative predictive value (NPV), and area under the receiver-operating characteristic curve (ROC) of individual antibodies (Abs), and combinations of Abs to classify participants as antigen-positive, Samoa 2018.

Serological indicator(s)	Ab-positive n (%)	Sensitivity (%)	Specificity (%)	PPV (%)	NPV (%)	ROC
**Individual antibodies**						
*Bm14* Ab	583 (15.4)	88.9 (81.7-93.9)	87.0 (85.8-88.0)	17.8 (14.8-21.2)	99.6 (99.3-99.8)	0.88
*Bm33* Ab	1659 (43.7)	94.9 (89.2-98.1)	57.9 (56.3-59.5)	6.7 (5.5-8.0)	99.7 (99.4-99.9)	0.76
*Wb123* Ab	987 (26.0)	89.7 (82.8-94.6)	76.0 (74.6-77.4)	10.6 (8.8-12.7)	99.6 (99.3-99.8)	0.83
**Combinations of antibodies**						
*Wb123* Ab or *Bm14* Ab	1128 (29.7)	93.2 (87.0-97.0)	72.3 (70.8-73.7)	9.7 (8-11.5)	99.7 (99.4-99.9)	0.83
*Wb123* Ab or *Bm33* Ab	1853 (48.8)	95.7 (90.3-98.6)	52.7 (51.0-54.3)	6.0 (5.0-7.2)	99.7 (99.4-99.9)	0.74
*Bm14* Ab or *Bm33* Ab	1707 (45.0)	97.4 (92.7-99.5)	56.7 (55.1-58.3)	6.7 (5.5-8)	99.9 (99.6-100.0)	0.77
Any Ab	1889 (57.8)	97.4 (92.7-99.5)	51.7 (50.1-53.4)	6.0 (5.0-7.2)	99.8 (99.5-100.0)	0.75
*Wb123* Ab and *Bm14* Ab	442 (11.7)	85.5 (77.8-91.3)	90.7 (89.7-91.6)	22.6 (18.8-26.8)	99.5 (99.2-99.7)	0.88
*Wb123* Ab and *Bm33* Ab	793 (20.9)	88.9 (81.7-93.9)	81.3 (80-82.5)	13.1 (10.8-15.7)	99.6 (99.3-99.8)	0.85
*Bm14* Ab and *Bm33* Ab	535 (14.1)	86.3 (78.7-92.0)	88.2 (87.1-89.2)	18.9 (15.6-22.5)	99.5 (99.2-99.7)	0.87
All Ab	430 (11.3)	84.6 (76.8-90.6)	91.0 (90-91.9)	23.0 (19.1-27.3)	99.5 (99.2-99.7)	0.88

Blue: 0-50.0%; green: 50.1-75.0%; yellow: 75.1-90.0%; orange: >90%. Ab = antibody, NPV = negative predictive value, PPV = positive predictive value, ROC = area under the Receiver-Operating Characteristic Curve.

### Associations between LF-seropositivity and age group and PSU selection

**Fig 4:**
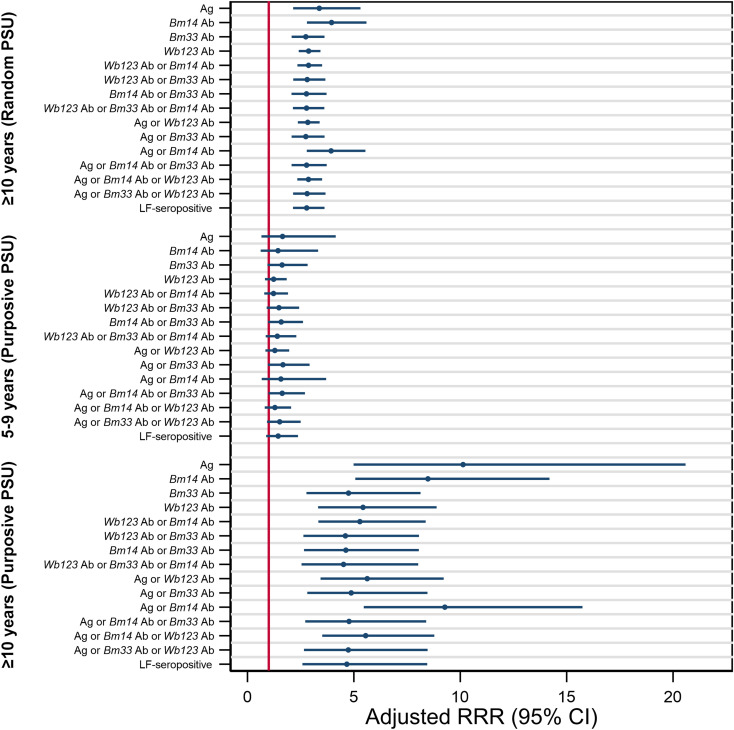
Adjusted relative risk ratio (RRR) for testing positive to antigen (Ag), antibody (Ab), and a combination of markers, by age and primary sampling unit (PSU) selection, compared with children aged 5-9 years as a reference, Samoa 2018. Red line indicates an RRR of 1.0. Values above 1.0 indicate an increased risk of seropositivity for the Ag, Ab, or combination of Ag and Ab listed on the y-axis. Values below 1.0 indicate a reduced risk of seropositivity.

The probability of testing positive for Ag, Ab, or a combination of Abs was significantly higher among participants aged ≥10 years from both randomly selected (*p*<0.001) and purposively selected PSUs (*p*<0.001), when compared to participants aged 5-9 years from randomly selected PSUs. The largest effect size was observed in participants aged ≥10 years from purposively selected PSUs, where the adjusted probability of testing positive for Ag (RRR=10.2), Ag and/or *Bm14* Ab (RRR=9.3), or *Bm14* Ab (RRR=8.5) was significantly higher (Fig 4 and S4 Table). Participants aged ≥10 years from purposive PSUs had at least twice the probability of testing positive to all seromarkers compared to participants ≥10 years from randomly selected PSUs; the largest effect size was observed for Ag (RRR=3.0), Ag or *Bm14* Ab (RRR=2.4), or *Bm14* Ab (RRR=2.2) (S6 Table and S5 Fig).

### Ratio of Ab-positives versus Ag-positives by age-group and PSU selection

**Fig 5:**
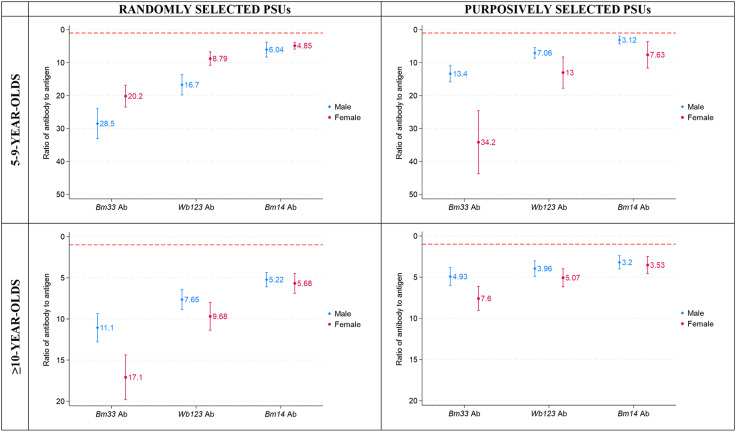
Ratio of antibody (Ab) to antigen (Ag)-positives for *Bm33*, *Wb123* and *Bm14* Abs by age, sex, and primary sampling unit (PSU) selection, adjusted for survey design, Samoa 2018. Dashed red line indicates a ratio of 1.0.

Ratios of Ab-positivity to Ag-positivity was investigated by age group, sex, and PSU selection. A high ratio of individuals tested positive for Ab only, suggesting LF-seropositives would be missed if Ag was used alone (Fig 4). The largest ratio was seen for *Bm33* Ab among females aged 5-9 years in purposively selected PSUs (34.2 [95% CI: 24.6-43.8]), followed by males (28.5 [95% CI: 23.9-33.1]) and females (20.2 [95% CI: 16.8-23.5]) 5-9 years old in randomly selected PSUs. The lowest ratio was seen for *Bm14* Ab among 5-9-year-old males (3.1 [95% CI: 2.0-4.3]), ≥10-year-old males (3.2 [95% CI: 2.4-4.0]), and ≥10-year-old females (3.5 [95% CI: 2.5-4.6]) in purposively selected PSUs (Fig 5).

### Number needed to test

For all subgroups, the number of participants needed to test to identify one positive participant (NNTest^av^) was lowest for *Bm33* Ab, followed by *Wb123* Ab, *Bm14* Ab and highest for Ag. For all seromarkers, the NNTest^av^ decreased as age increased. Similarly, NNTest^av^ was lower in purposively selected PSUs compared to randomly selected PSUs (Table 2).

**Table 2:** Number need to test for different sampling strategies and age groups using antigen (Ag), *Bm14* antibody (Ab)*, Wb123* Ab, and *Bm33* Ab, Samoa 2018. (A) Average number needed to test to identify one positive person (NNTest^av^), (B) Number needed to test to provide a 50% chance of identifying at least one positive person (NNTest^50^), and (C) Number needed to test to provide a 95% chance of identifying at least one positive person (NNTest^95^).

(A) NNTest^av^	Age (years)	*Bm14* Ab	*Bm33* Ab	*Wb123* Ab	Ag	Legend
**Randomly selected PSUs**	**5-9**	14.6	3.3	6.4	78.5	<5
	**10-19**	9.0	2.7	4.5	63.6	5-9.9
	**20-39**	4.2	1.8	2.6	23.0	10-24.9
	**40-59**	3.2	1.5	2.5	14.2	25-49.9
	**≥60**	3.9	1.6	2.7	26.9	50-99.9
	**≥10**	4.5	1.8	2.9	24.3	≥100
**Purposively selected PSUs**	**5-9**	10.6	2.4	5.4	48.8	
	**10-19**	3.8	2.0	2.7	27.7	
	**20-39**	1.8	1.3	1.5	7.1	
	**40-59**	2.6	1.3	1.8	6.1	
	**≥60**	2.9	1.3	2.3	5.3	
	**≥10**	2.6	1.5	2.0	8.8	
**(B) NNTest** ^**50**^	**Ag (years)**	***Bm14* Ab**	***Bm33* Ab**	***Wb123* Ab**	**Ag**	
**Randomly selected PSUs**	**5-9**	9.8	1.9	4.1	54.1	
	**10-19**	5.8	1.5	2.7	43.7	
	**20-39**	2.6	0.8	1.4	15.6	
	**40-59**	1.8	0.6	1.3	9.5	
	**≥60**	2.4	0.7	1.5	18.3	
	**≥10**	2.8	0.9	1.7	16.5	
**Purposively selected PSUs**	**5-9**	6.9	1.3	3.4	33.4	
	**10-19**	2.3	1.0	1.5	18.9	
	**20-39**	0.8	0.4	0.6	4.5	
	**40-59**	1.5	0.5	0.9	3.8	
	**≥60**	1.6	0.4	1.2	3.3	
	**≥10**	1.5	0.6	1.0	5.7	
**(C) NNTest** ^**95**^	**Ag (years)**	***Bm14* Ab**	***Bm33* Ab**	***Wb123* Ab**	**Ag**	
**Randomly selected PSUs**	**5-9**	42.3	8.3	17.7	233.7	
	**10-19**	25.4	6.6	11.8	188.9	
	**20-39**	11.0	3.6	6.0	67.4	
	**40-59**	7.9	2.7	5.7	40.9	
	**≥60**	10.2	3.0	6.4	79.0	
	**≥10**	2.8	0.9	1.6	16.5	
**Purposively selected PSUs**	**5-9**	30.3	5.6	14.7	144.5	
	**10-19**	10.0	4.2	6.4	81.5	
	**20-39**	3.6	1.9	2.7	19.6	
	**40-59**	6.2	2.1	3.8	16.6	
	**≥60**	7.1	1.9	5.2	14.3	
	**≥10**	1.5	0.6	1.0	5.7	

**Fig 6:**
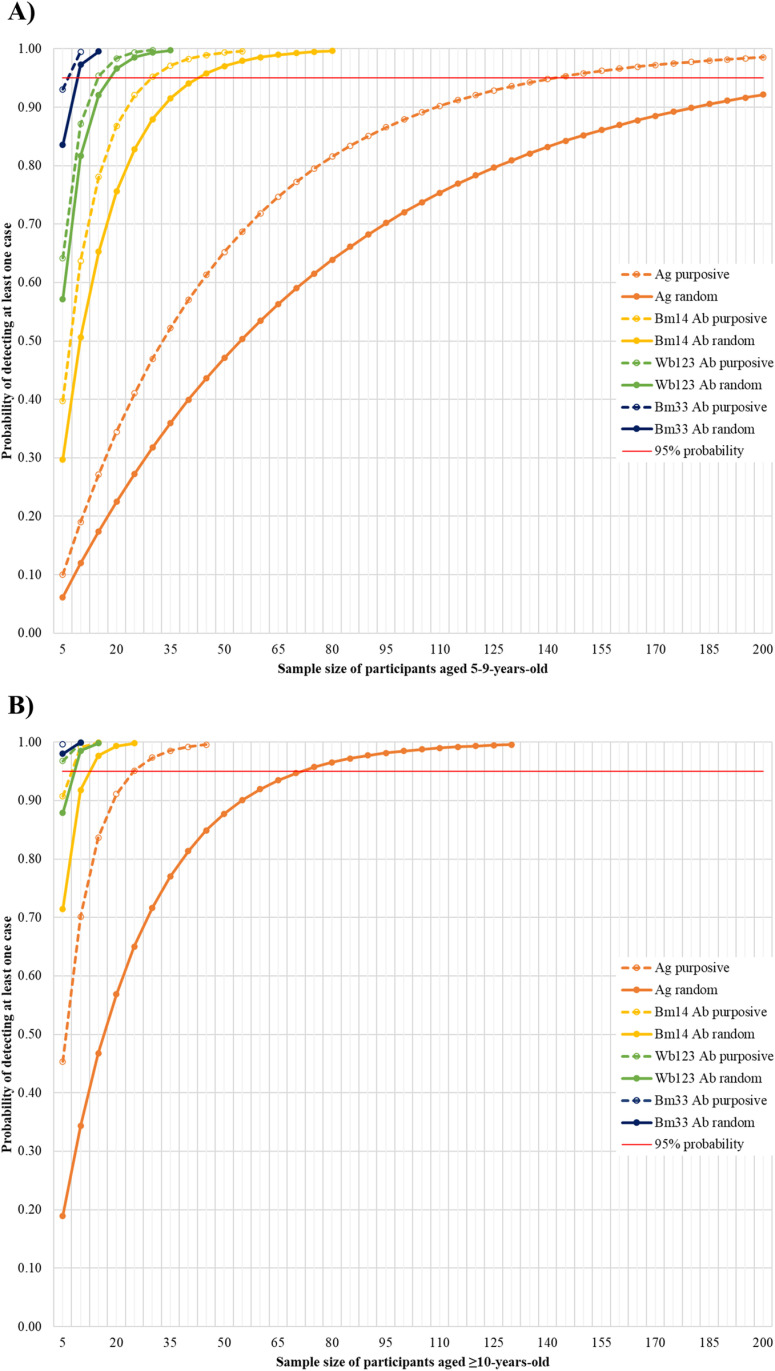
Probability of detecting at least one positive case of antigen (Ag), *Bm14* antibody (Ab), *Wb123* Ab, or *Bm33* Ab for (A) 5-9 years old in randomly and purposively selected primary sampling units (PSUs) and (B) ≥10 years old in randomly and purposively selected PSUs (assumes that positive cases are independent events).

Fig 6 shows the probability of at least one participant aged 5-9 years or ≥10 years testing positive for each seromarker based on the NNTest^av^ in Table 2. Based on the observed seroprevalence in 5-9-year-olds in randomly selected PSUs (1.3%), over 200 children aged 5-9 years would need to be tested to provide >95% probability of detecting at least one Ag-positive case. In contrast, only 45, 20, and 10 children aged 5-9 years would need to be tested to provide >95% probability of detecting at least one *Bm14* Ab-positive, *Wb123* Ab-positive, or *Bm33* Ab-positive case, respectively. In purposively selected PSUs, the NNTest^av^ dropped to at least 140 for one Ag-positive case, 30 for one *Bm14* Ab-positive case, 15 for one *Wb123* Ab-positive case, and 10 for one *Bm33* Ab-positive case.

When restricted to testing participants ≥10 years old, only 70, 15, 10, and five participants from randomly selected PSUs would need to be tested to identify at least one Ag-positive, *Bm14* Ab-positive, *Wb123* Ab-positive, or *Bm33* Ab-positive case, respectively. Among purposively selected PSUs, the NNTest^av^ dropped to 25, 10, and five participants to identify at least one Ag-positive, *Bm14* Ab-positive, or *Wb123* Ab-positive case. Only five participants in purposively selected PSUs would need to be tested to have close to 100% probability of detecting at least one *Bm33* Ab-positive case.

## Discussion

As more countries near LF elimination, sensitive and specific diagnostic tools are required to identify residual LF infections and enable timely intervention to prevent disease resurgence. This study has provided evidence on the potential value of using Abs as an adjunct to Ag for LF surveillance programs, supporting previous studies suggesting that Abs could be more sensitive indicators of LF infection than Ag alone [11]. Our study has quantified the proportion of LF-seropositive individuals that may be missed if Ag were used alone, as well as those missed when individual Abs and different combinations of Ab and Ag are used. We found that *Bm14* Ab had the highest ROC of the three Ab seromarkers to classify participants as Ag-positive, which was equal to testing positive for both *Wb123* Ab and *Bm14* Ab, or testing positive for all three Abs. This information is important for supporting WHO to select the most sensitive and specific diagnostic tools for LF surveillance.

Identifying asymptomatic individuals and residual foci of infection becomes increasingly challenging as LF prevalence decreases following successful MDA. The current target product profile for diagnostic tests for LF surveillance aims for a minimum sensitivity of 85% and specificity of 98.8% [26]. In our study, we used Ag-positivity as a reference for assessing the sensitivity and specificity of Abs because Ag is currently the main indicator used for LF surveillance and programmatic decisions. We found that all Abs and combinations of Abs had a sensitivity >85%, but no individual Ab, or combination of Abs, had a specificity >98.8%. We acknowledge the limitations of using Ag-positivity as a reference, as Ag can persist for months following effective treatment [27] and being Ag-positive does not always indicate active infection. Further, due to the timing of our survey immediately following MDA, we were unable to use Mf data to identify infectious patients in our sample.

Of the three Ab seromarkers, *Bm33* Ab had the lowest ROC to classify participants as Ag-positive. The high seroprevalence of *Bm33* Ab in previous surveys [12,20] suggests it is very long-lasting post-infection. Thus, the poor discriminatory value of *Bm33* Ab limits its use for LF surveillance in programmatic surveys, particularly in endemic areas with previously high infection rates. Contrastingly, we found *Bm14* Ab had the highest ROC of the three Abs, but the lowest sensitivity to detect Ag-positives. Though the higher ROC of *Bm14* Ab is promising, it may not be sufficiently sensitive to support LF surveillance alone, but could be useful if tested in combination with Ag. The ROC of *Wb123* Ab was between that of *Bm14* Ab and *Bm33* Ab, suggesting *Wb123* Ab may be more discriminative than *Bm33* Ab to identify true positives and more sensitive than *Bm14* Ab. This finding is supported by a study in Papua New Guinea that found *Wb123* Ab responses were more closely correlated to persistent Mf infection compared to Ag and *Bm14* Ab responses [28]. A further 2015 study of Togolese school children who were born shortly before or after MDA was discontinued found that 4.7% were *Wb123* Ab-positive, despite recent surveys finding very few Ag-positive or Mf-positive cases [29].

An investigation of Ab responses in three sequential TAS in American Samoa reported that seropositivity to a combination of *Wb123* Ab and *Bm14* Ab, or any of *Bm14* Ab, *Wb123*, or *Bm33* Ab in TAS-2 were highly sensitive and specific for predicting schools with Ag-positive children in the following survey [12]. Our study found that testing positive to both *Wb123* Ab and *Bm14* Ab had a sensitivity >85% and specificity >90%, suggesting that testing for both *Wb123* Ab and *Bm14* Ab, possibly alongside Ag, could be an approach to improve LF surveillance. Indeed, studies have found that Ag-positive and Mf-positive children are generally seropositive for at least two Abs [30].

As countries approach elimination and enter the Stop MDA Phase, WHO recommends conducting TAS to estimate LF Ag prevalence among 6-7-year-old children to determine whether the critical threshold of <1% Ag prevalence (in areas where *W. bancrofti* is endemic and *Aedes* is the primary vector [31]) has been reached. However, our study found that i) the probability of testing positive for all seromarkers was significantly higher among ≥10-year-olds compared to 5-9-year-olds; ii) the NNTest^av^ was significantly lower among ≥10-year-olds compared to 5-9-year-olds; and iii) the correlation between seromarker prevalence at the PSU level was weakest when restricted to 5-9-year-olds compared to ≥10-year-olds. Given our findings, conducting TAS among older aged school children and/or among community members, particularly in suspected hotspots, may increase the likelihood of detecting persistent LF transmission and could prevent the premature cessation of MDA and other surveillance activities.

Interestingly, we found that the ratio of Ab-positivity to Ag-positivity was largest in female participants aged 5-9 years in purposively selected PSUs, and among participants aged ≥10 years in both purposively selected and randomly selected PSUs, suggesting that there might be gender differences in Ab responses, and therefore the utility of Abs for surveillance. Previous studies have found that females elicited *Bm14* Ab responses at an earlier age than males [11]. It would be interesting to investigate whether Ab responses are influenced by gender, and if so, how this may impact LF surveillance moving forward.

The serological course and duration of persistence of LF Abs is poorly understood and more research is needed to understand anti-filarial Ab kinetics. Indeed, longitudinal monitoring has shown that individual Abs are detected at different times over the course of infection and their expression can differ by age [11]. Further, population-level Ab prevalence has been seen to drop after MDA [5,32,33], but Abs can persist for a long time following clearance of Ag from the original infection (though the exact duration is uncertain) [14,15]. Thus, Ab-positivity is not always indicative of active infection [15]. This can have cost implications for national programs if Ab-positive individuals are treated unnecessarily. Further research may aid in the interpretation of Ab signals and elucidate whether infection can be classified as active or past based on serological patterns of Ab alone.

Despite these knowledge gaps, there are advantages of Ab serosurveillance. Firstly, there is a long latency period between LF infection and the development of antigenemia (Ag tests may take months to become positive after infection) [29]. By contrast, Ab responses develop before patent infection or antigenemia, and antigenemia is not a requirement for robust Ab responses [11]. Longitudinal monitoring of Abs in Haiti suggest that *Bm33* Ab may be a marker of very early LF infection; this study found that *Bm33* was the first Ag to elicit an Ab response and was detected on average more than one year before other Ab responses [11]. *Bm14* Ab and *Wb123* Ab responses developed after *Bm33* Ab, but on average six months before the development of antigenemia [11].

Secondly, the high sensitivity of Ab compared to Ag is a particular asset in the context of LF endgame strategies and may enable the identification and management of LF hotspots and residual transmission foci. Spatial analysis using Ag and Ab seroprevalence estimates in American Samoa found that clusters of Ab seropositives were strongly correlated with the presence of multiple Mf-positives, indicating the potential utility of Ab serosurveillance to identify ongoing transmission [34].

Thirdly, there is the potential for monitoring seroprevalence for different seromarkers at different stages of the elimination program. For example, Abs that are rapidly cleared (such as *Bm14* Ab and *Wb123* Ab) could be monitored in countries in the Stop MDA Phase, whilst less specific but highly sensitive Abs (such as *Bm33* Ab) could be used in elimination settings among populations that should be immunologically naïve with no prior LF exposure. This could aid in identifying cut-off measures for more recent exposure or active infection [35,36]. Lastly, technologies such as MBA only require a small volume of blood collected on filter paper to detect Abs, which is logistically and resourcefully appealing. MBA have the added advantage of simultaneously detecting Ab to other diseases of interest, such as vaccine-preventable diseases, arboviruses, and other NTDs, that can enable integrated disease surveillance, management, and treatment.

In conclusion, as more countries approach elimination, further work is urgently needed to determine how Ab serosurveillance can be used to support national programs. Abs are more sensitive than Ag for identifying LF-seropositive participants and may be a useful tool for LF surveillance and elimination programmes. Here, we have established baseline Ab and Ag seroprevalence estimates in Samoa that can be monitored longitudinally to investigate the impact of MDA and further investigate the sensitivity of Ab compared to Ag over time. Further, the low specificity of Abs means that further research is required to fully understand the behaviour of each Ab to determine how Ab serosurveillance can best be used to support LF surveillance programmes.

## Supplementary information

S1 Table Demographics of study population in randomly versus purposively selected PSUs, Samoa 2018. (DOCX)

S2 Table Antibody (Ab) and antigen (Ag) prevalence by age (adjusted for sampling design and standardised by sex) and primary sampling unit (PSU) (adjusted for survey design and standardised by age and sex), Samoa 2018. Significantly more participants aged ≥5 years from purposively selected PSUs were seropositive for Ag, *Bm14* Ab, *Bm33* Ab, and *Wb123* Ab compared to randomly selected PSUs. Adjusted prevalence for all infection markers were significantly lower in participants aged 5–9 years compared to those aged ≥10 years. Significantly more participants aged ≥10 years from purposively selected PSUs were seropositive for Ag, *Bm14* Ab, *Bm33* Ab, and *Wb123* Ab compared to participants from randomly selected PSUs.
(DOCX)

S3 Table Proportion of LF-seropositives that would be ‘missed’ by individual, and combinations of, seromarkers, adjusted for sampling design and standardised by age and sex, Samoa 2018. Most LF-seropositives would have been ‘missed’ if tested for antigen alone. The least LF-positives would have been missed if tested for *Bm33* antibody (Ab) and/or *Wb123* Ab.
(DOCX)

S4 Table Serological profile of participants testing negative to individual, and combinations, of seromarkers, adjusted for sampling design and standardised by age and sex, Samoa 2018. (DOCX)

S5 Table Sensitivity, specificity, positive predictive value (PPV), negative predictive value (NPV), and area under the Receiver-Operating Characteristic Curve (ROC) of individual antibodies (Abs), and combinations of Abs to classify participants as antigen-positive among 5-9-year-olds and ≥10-year-olds, Samoa 2018. (DOCX)

S6 Table Unadjusted and adjusted multinomial logistic regression analysis for testing positive to different antigen (Ag) and antibody (Ab) combinations by age group and sampling design in relation to participants aged 5–9 years old in randomly selected primary sampling units (PSU), Samoa 2018. Colour spectrum from blue to yellow to green indicates progressive increase in risk. Light blue denotes low relative risk ratio (RRR), dark green denotes high RRR.
(DOCX)

S7 Table Unadjusted and adjusted multinomial logistic regression analysis for testing positive to different antigen (Ag) and antibody (Ab) combinations among participants aged ≥10 years old and primary sampling unit (PSU) selection, Samoa 2018. (DOCX)

S1 Fig Flow chart of participant inclusion and exclusion, Samoa 2018. (DOCX)

S2 Fig Pearson’s correlation coefficient estimates for the relationship between antigen (Ag) prevalence and antibody (Ab) prevalence in participants aged ≥5 years, 5-9 years and ≥10 years at the primary sampling unit (PSU) level, Samoa 2018. (DOCX)

S3 Fig Pearson’s correlation coefficient estimates for the relationship between antibody (Ab) prevalence in participants aged ≥5 years, 5-9 years and ≥10 years at the primary sampling unit (PSU) level, Samoa 2018. (DOCX)

S4 Fig Sensitivity, specificity, and area under the Receiver-Operating Characteristic Curve (ROC) of antigen (Ag), antibodies (Abs), and combinations of Abs for 5-9-year-olds (TOP) and ≥10-year-olds (BOTTOM), Samoa 2018. (DOCX)

S5 Fig Adjusted relative risk ratio (RRR) of testing positive to antigen (Ag), antibody (Ab), and a combination of markers, among participant’s aged ≥10 years old from purposively selected primary sampling units (PSUs), using participants ≥10 years old from randomly selected PSUs as the referent, Samoa 2018. Values above 1.0 indicate and increased risk of seropositivity for the Ag, Ab, or combination of Ag and Ab listed on the Y-axis. Vales below 1.0 indicate a reduced risk of seropositivity.
(DOCX)
