# Supplementary material for: Sensitivity of anti-filarial antibodies for lymphatic filariasis surveillance: Insights from a serological survey in Samoa in 2018
Source: PLoS Negl Trop Dis. 2025 Jan 30;19(1):e0012835. doi: 10.1371/journal.pntd.0012835 (PMC11922241; doi:10.1371/journal.pntd.0012835)
Supplement: S1 Table — (DOCX) [file pntd.0012835.s001.docx]

**Supplementary Table 1: Demographics of study population in randomly versus purposively selected PSUs, Samoa 2018.**

| **Variable** | **All participants**  **N=3795** | **Randomly selected PSU**  **N=3277** | **Purposively selected PSU**  **N=518** | ***P*-value** |
| --- | --- | --- | --- | --- |
| Age (years [SD]) | 20.7±19.1 | 20.8±19.2 | 20.0±18.6 | 0.39 |
| Age group (years) |  |  |  |  |
| 5-9 | 1896 (50.0) | 1641 (50.1) | 255 (49.2) | 0.72 |
| ≥10 | 1899 (50.0) | 1636 (49.9) | 263 (50.8) |  |
| Sex |  |  |  |  |
| Female | 1942 (51.2) | 1667 (50.9) | 275 (53.1) | 0.35 |
| Male | 1853 (48.8) | 1610 (49.1) | 243 (46.9) |  |
| Survey type |  |  |  |  |
| Household | 2283 (60.2) | 1970 (60.1) | 313 (60.4) | 0.89 |
| Convenience | 1512 (39.8) | 1307 (39.9) | 205 (39.6) |  |
| Household size (years) | 6.7±4.0 | 6.6±4.1 | 6.8±3.1 | 0.42 |
| Took MDA in 2018 |  |  |  |  |
| No | 349 (9.2) | 297 (9.1) | 52 (10.1) | 0.47 |
| Yes | 3441 (90.8) | 2976 (90.9) | 465 (89.9) |  |
| Taken MDA in the past |  |  |  |  |
| No | **1325 (35.0)** | **1225 (37.4)** | **100 (19.3)** | **<0.001** |
| Yes | **2466 (65.0)** | **2049 (62.6)** | **417 (80.7)** |  |
| Know about MDA |  |  |  |  |
| No | 280 (7.4) | 237 (7.2) | 43 (8.3) | 0.39 |
| Yes | 3515 (92.6) | 3040 (92.8) | 475 (91.7) |  |
| Time lived in Samoa |  |  |  |  |
| Less than whole life | **408 (10.8)** | **382 (11.7)** | **26 (5.0)** | **<0.001** |
| Whole life | **3387 (89.2)** | **2895 (88.3)** | **492 (95.0)** |  |
| Region |  |  |  |  |
| Apia Urban Area | **644 (17.0)** | **644 (19.7)** | **0 (0.0)** | **<0.001** |
| Northwest Upolu | **1552 (40.9)** | **1212 (37.0)** | **340 (65.6)** |  |
| Rest of Upolo | **870 (22.9)** | **798 (24.4)** | **72 (13.9)** |  |
| Savai’i | **729 (19.2)** | **623 (19.0)** | **106 (20.5)** |  |

*Data are presented as number (%) or mean ± standard deviation.*
