# Supplementary material for: Sensitivity of anti-filarial antibodies for lymphatic filariasis surveillance: Insights from a serological survey in Samoa in 2018
Source: PLoS Negl Trop Dis. 2025 Jan 30;19(1):e0012835. doi: 10.1371/journal.pntd.0012835 (PMC11922241; doi:10.1371/journal.pntd.0012835)
Supplement: S2 Table — Adjusted prevalence for all infection markers were significantly lower in participants aged 5–9 years compared to those aged ≥10 years. Significantly more participants aged ≥10 years from purposively selected PSUs were seropositive for Ag, Bm14 Ab, Bm33 Ab, and Wb123 Ab compared to participants from randomly selected PSUs. (DOCX) [file pntd.0012835.s002.docx]

**Supplementary Table 2: Antibody (Ab) and antigen (Ag) prevalence by age (adjusted for sampling design and standardised by sex) and primary sampling unit (PSU) (adjusted for survey design and standardised by age and sex), Samoa 2018.** Significantly more participants aged ≥5 years from purposively selected PSUs were seropositive for Ag, *Bm14* Ab, *Bm33* Ab, and *Wb123* Ab compared to randomly selected PSUs. Adjusted prevalence for all infection markers were significantly lower in participants aged 5-9 years compared to those aged ≥10 years. Significantly more participants aged ≥10 years from purposively selected PSUs were seropositive for Ag, *Bm14* Ab, *Bm33* Ab, and *Wb123* Ab compared to participants from randomly selected PSUs.

|  | **Overall** | | | **Purposively selected PSUs** | | | | | | | | | **Randomly selected PSUs** | | | | | | | | |  |  |  |
| --- | --- | --- | --- | --- | --- | --- | --- | --- | --- | --- | --- | --- | --- | --- | --- | --- | --- | --- | --- | --- | --- | --- | --- | --- |
|  | **Total testing positive^** | | | **Total testing positive^** | | | **Age 5-9 years old  testing positive*** | | | **Age ≥10 years old**  **testing positive*** | | | **Total testing positive^** | | | **Age 5-9 years old**  **testing positive*** | | | **Age ≥10 years old**  **testing positive*** | | | ***p-value 1*** | ***p-value 2*** | ***p-value 3*** |
|  | **N** | **%** | **95% CI** | **N** | **%** | **95% CI** | **N** | **%** | **95% CI** | **N** | **%** | **95% CI** | **N** | **%** | **95% CI** | **N** | **%** | **95% CI** | **N** | **%** | **95% CI** |  |  |  |
| Total | 3795 |  |  |  |  |  |  |  |  |  |  |  |  |  |  |  |  |  |  |  |  |  |  |  |
| Ag | 117 | 3.7 | (2.6, 5.2) | 31 | 10.0 | (7.4, 13.3) | 4 | 2.1 | (1.0, 4.3) | 27 | 11.4 | (7.9, 16.1) | 86 | 3.5 | (2.4, 5.0) | 24 | 1.3 | (0.8, 2.1) | 62 | 4.1 | (2.7, 6.3) | **<0.001** | 0.276 | **0.001** |
| *Bm14* Ab | 583 | 20.3 | (16.9, 24.3) | 125 | 36.4 | (31.9, 41.1) | 24 | 9.4 | (4.9, 17.4) | 101 | 37.8 | (30.5, 45.7) | 458 | 19.8 | (16.3, 23.9) | 123 | 6.8 | (4.8, 9.7) | 335 | 22.2 | (17.1, 28.2) | **<0.001** | 0.380 | **0.003** |
| *Bm33* Ab | 1659 | 51.0 | (45.5, 56.6) | 297 | 66.6 | (57.1, 74.8) | 111 | 41.3 | (30.3, 53.3) | 186 | 67.2 | (56.8, 76.2) | 1362 | 50.5 | (44.8, 56.2) | 509 | 30.3 | (24.8, 36.5) | 853 | 54.3 | (47.2, 61.2) | **<0.001** | 0.083 | **0.048** |
| *Wb123* Ab | 987 | 32.2 | (26.6, 38.3) | 184 | 47.9 | (42.4, 53.4) | 49 | 18.5 | (15.8, 21.4) | 135 | 49.7 | (41.5, 57.9) | 803 | 31.7 | (25.9, 38.0) | 265 | 15.6 | (11.6, 20.7) | 538 | 34.4 | (27.3, 42.3) | **<0.001** | 0.304 | **0.012** |
| Any Ab | 1889 | 57.8 | (51.8, 63.6) | 317 | 70.7 | (61.1, 78.7) | 119 | 44.4 | (34.3, 55.0) | 198 | 72.0 | (61.4, 80.6) | 1572 | 57.4 | (51.2, 63.4) | 608 | 36.4 | (30.5, 42.6) | 964 | 61.2 | (53.5, 68.4) | **<0.001** | 0.178 | 0.107 |
| LF-seropositive | 1892 | 57.9 | (51.8, 63.7) | 319 | 71.7 | (61.3, 80.2) | 120 | 45.1 | (34.9, 55.8) | 199 | 72.8 | (61.8, 81.6) | 1573 | 57.4 | (51.2, 63.4) | 608 | 36.4 | (30.5, 42.6) | 965 | 61.3 | (53.3, 68.5) | **<0.001** | 0.148 | 0.092 |

**Adjusted for sampling design and standardised by sex; ^adjusted for survey design and standardised by age and sex. Ag: Antigen; Ab: Antibody; CI: Confidence Interval; PSU: Primary Sampling Unit.*

*p-*value 1: Testing for significant differences in prevalence between participants 5-9 years old and ≥10 years old; *p*-value 2: Testing for significant differences in prevalence between participants 5-9 years old in randomly and purposively selected PSUs; *p-*value 3: Testing for significant differences in prevalence between participants ≥10 years old in randomly and purposively selected PSUs.
