# Supplementary material for: Sensitivity of anti-filarial antibodies for lymphatic filariasis surveillance: Insights from a serological survey in Samoa in 2018
Source: PLoS Negl Trop Dis. 2025 Jan 30;19(1):e0012835. doi: 10.1371/journal.pntd.0012835 (PMC11922241; doi:10.1371/journal.pntd.0012835)
Supplement: S3 Table — The least LF-positives would have been missed if tested for Bm33 antibody (Ab) and/or Wb123 Ab. (DOCX) [file pntd.0012835.s003.docx]

**Supplementary Table 3: Proportion of LF-seropositives that would be ‘missed’ by individual, and combinations of, seromarkers, adjusted for sampling design and standardised by age and sex, Samoa 2018.** Most LF-seropositives would have been ‘missed’ if tested for antigen (Ag) alone. The least LF-positives would have been missed if tested for *Bm33* antibody (Ab) and/or *Wb123* Ab.

|  | **Total LF-seropositives ‘missed’** | | |
| --- | --- | --- | --- |
|  | **N** | **%** | **95% CI** |
| **Total** | **1892** |  |  |
| **Seronegative to individual antibodies** | | | |
| Ag-negative | 1775 | 94.1 | (92.1-95.6) |
| *Bm14* Ab-negative | 1309 | 67.3 | (63.1-71.3) |
| *Wb123* Ab-negative | 905 | 45.9 | (39.8-52.2) |
| *Bm33* Ab-negative | 233 | 12.6 | (10.4-15.2) |
| **Seronegative to combinations of antibodies and antigen** | | | |
| *Bm14* Ab-negative or Ag-negative | 1296 | 66.9 | (62.7-70.9) |
| *Wb123* Ab-negative or Ag-negative | 893 | 45.6 | (48.1-60.6) |
| *Bm14* Ab-negative or *Wb123* Ab-negative | 764 | 38.9 | (32.7-45.5) |
| *Bm33* Ab-negative or Ag-negative | 227 | 12.4 | (10.1-15.1) |
| *Bm14* Ab-negative or *Bm33* Ab-negative | 185 | 9.8 | (7.8-12.1) |
| *Bm33* Ab-negative or *Wb123* Ab-negative | 39 | 1.9 | (1.1-3.2) |

*Ag: Antigen; Ab: Antibody; CI: Confidence Interval.*
