# Supplementary material for: Sensitivity of anti-filarial antibodies for lymphatic filariasis surveillance: Insights from a serological survey in Samoa in 2018
Source: PLoS Negl Trop Dis. 2025 Jan 30;19(1):e0012835. doi: 10.1371/journal.pntd.0012835 (PMC11922241; doi:10.1371/journal.pntd.0012835)
Supplement: S4 Table — (DOCX) [file pntd.0012835.s004.docx]

**Supplementary Table 4: Serological profile of participants testing negative to individual, and combinations, of seromarkers, adjusted for sampling design and standardised by age and sex, Samoa 2018.**

|  | **Seroprevalence** | | |
| --- | --- | --- | --- |
|  | **N** | **%** | **95% CI** |
| **Ag-negative (n=3678)** | | | |
| Any Ab-positive | 1775 | 56.5 | 50.6-62.3 |
| *Bm14* Ab-positive | 479 | 17.7 | 14.6-21.3 |
| *Wb123* Ab-positive | 882 | 29.9 | 24.5-35.9 |
| *Bm33* Ab-positive | 1548 | 49.5 | 44.2-54.9 |
| ***Bm14* Ab-negative (n=3212)** | | | |
| Ag-positive | 13 | 0.3 | 0.1-0.7 |
| *Wb123* Ab-positive | 545 | 20.9 | 16.3-26.4 |
| *Bm33* Ab-positive | 1124 | 42.0 | 37.0-47.1 |
| ***Wb123* Ab-negative (n=2802)** | | | |
| Ag-positive | 12 | 0.2 | 0.1-0.6 |
| *Bm14* Ab-positive | 141 | 6.9 | 5.0-9.6 |
| *Bm33* Ab-positive | 866 | 37.7 | 32.2-43.6 |
| ***Bm33* Ab-negative (n=2136)** | | | |
| Ag-positive | 6 | 0.2 | 0.1-0.7 |
| *Bm14* Ab-positive | 48 | 3.8 | 2.4-6.1 |
| *Wb123* Ab-positive | 194 | 11.8 | 8.6-15.9 |
| ***Bm14* Ab-negative or Ag-negative (n=3199)** | | | |
| *Wb123* Ab-positive | 540 | 20.9 | 16.3-26.3 |
| *Bm33* Ab-positive | 1114 | 41.9 | 37.0-47.1 |
| ***Wb123* Ab-negative or Ag-negative** | | | |
| *Bm14* Ab-positive | 137 | 6.9 | 4.9-9.5 |
| *Bm33* Ab-positive | 859 | 37.7 | 32.1-43.6 |
| ***Bm33* Ab-negative or Ag-negative** | | | |
| *Bm14* Ab-positive | 45 | 3.7 | 2.3-6.0 |
| *Wb123* Ab-positive | 137 | 11.8 | 8.6-15.9 |
| ***Bm14* Ab-negative or *Wb123* Ab-negative** | | | |
| Ag-positive | 8 | 0.2 | 0.0-0.6 |
| *Bm33* Ab-positive | 761 | 35.2 | 29.8-41.0 |
| ***Bm14* Ab-negative or *Bm33* Ab-negative** | | | |
| Ag-positive | 3 | 0.1 | 0.0-0.7 |
| *Wb123* Ab-positive | 182 | 10.9 | 8.2-14.4 |
| ***Bm33* Ab-negative or *Wb123* Ab-negative (n=1942)** | | | |
| Ag-positive | 5 | 0.2 | 0.1-0.7 |
| *Bm14* Ab-positive | 36 | 2.8 | 1.7-4.8 |

*Ag: Antigen; Ab: Antibody; CI: Confidence Interval.*
