# Supplementary material for: Sensitivity of anti-filarial antibodies for lymphatic filariasis surveillance: Insights from a serological survey in Samoa in 2018
Source: PLoS Negl Trop Dis. 2025 Jan 30;19(1):e0012835. doi: 10.1371/journal.pntd.0012835 (PMC11922241; doi:10.1371/journal.pntd.0012835)
Supplement: S5 Table — (DOCX) [file pntd.0012835.s005.docx]

**Supplementary Table 5: Sensitivity, specificity, positive predictive value (PPV), negative predictive value (NPV), and area under the Receiver-Operating Characteristic Curve (ROC) of individual antibodies (Abs), and combinations of Abs to classify participants as antigen-positive among 5-9-year-olds and ≥10-year-olds, Samoa 2018.**

| **Indicator** | **Ag positive  (n)** | **Ab positive  (n)** | **Chi^2^** | ***p*-value** | **Sensitivity (%)** | **Specificity (%)** | **PPV (%)** | **NPV (%)** | **ROC** |
| --- | --- | --- | --- | --- | --- | --- | --- | --- | --- |
| **5-9-year-olds** | | | | | | | | | |
| **Individual Ab** | | | | | | | | | |
| *Bm14* Ab | 28 | 147 | 219.89 | <0.001 | 82.1 (63.1-93.9) | 93.4 (92.1-94.4) | 15.6 (10.2-22.5) | 99.7 (99.3-99.9) | 0.88 |
| *Bm33* Ab | 28 | 620 | 46.73 | <0.001 | 92.9 (76.5-99.1) | 68.2 (66.0-70.3) | 4.2 (2.8-6.1) | 99.8 (99.4-100) | 0.81 |
| *Wb123* Ab | 28 | 314 | 70.24 | <0.001 | 75.0 (55.1-89.3) | 84.3 (82.6-85.9) | 6.7 (4.2-10.0) | 99.6 (99.1-99.8) | 0.80 |
| **Combinations of Ab** | | | | | | | | | |
| *Wb123* Ab or *Bm14* Ab | 28 | 371 | 79.01 | <0.001 | 85.7 (67.3-96) | 81.4 (79.6-83.2) | 6.5 (4.2-9.5) | 99.7 (99.3-99.9) | 0.84 |
| *Wb123* Ab or *Bm33* Ab | 28 | 711 | 37.16 | <0.001 | 92.9 (76.5-99.1) | 63.3 (61.1-65.5) | 3.7 (2.4-5.3) | 99.8 (99.4-100) | 0.78 |
| *Bm14* Ab or *Bm33* Ab | 28 | 640 | 49.92 | <0.001 | 96.4 (81.7-99.9) | 67.2 (65-69.3) | 4.2 (2.8-6.1) | 99.9 (99.6-100) | 0.82 |
| Any Ab | 28 | 727 | 40.56 | <0.001 | 96.4 (81.7-99.9) | 62.5 (60.3-64.7) | 3.7 (2.5-5.4) | 99.9 (99.5-100) | 0.79 |
| *Wb123* Ab and *Bm14* Ab | 28 | 90 | 279.48 | <0.001 | 71.4 (51.3-86.8) | 96.3 (95.3-97.1) | 22.2 (14.1-32.2) | 99.6 (99.1-99.8) | 0.84 |
| *Wb123* Ab and *Bm33* Ab | 28 | 223 | 109.51 | <0.001 | 75.0 (55.1-89.3) | 89.2 (87.7-90.6) | 9.4 (5.9-14.0) | 99.6 (99.1-99.8) | 0.82 |
| *Bm14* Ab and *Bm33* Ab | 28 | 127 | 234.91 | <0.001 | 78.6 (59.0-91.7) | 94.4 (93.2-95.4) | 17.3 (11.2-25.0) | 99.7 (99.3-99.9) | 0.86 |
| All Ab | 28 | 86 | 293.68 | <0.001 | 71.4 (51.3-86.8) | 96.5 (95.5-97.3) | 23.3 (14.8-33.6) | 99.6 (99.1-99.8) | 0.84 |
| **≥10-year-olds** | | | | | | | | | |
| **Individual Ab** | | | | | | | | | |
| *Bm14* Ab | 89 | 436 | 244.48 | <0.001 | 91.0 (83.1-96.0) | 80.4 (78.5-82.2) | 18.6 (15.0-22.6) | 99.5 (98.9-99.8) | 0.86 |
| *Bm33* Ab | 89 | 1039 | 62.71 | <0.001 | 95.5 (88.9-98.8) | 47.3 (45.0-49.6) | 8.2 (6.6-10) | 99.5 (98.8-99.9) | 0.71 |
| *Wb123* Ab | 89 | 673 | 141.79 | <0.001 | 94.4 (87.4-98.2) | 67.5 (65.2-69.6) | 12.5 (10.1-15.2) | 99.6 (99.1-99.9) | 0.81 |
| **Combinations of Ab** | | | | | | | | | |
| *Wb123* Ab or *Bm14* Ab | 89 | 757 | 120.6 | <0.001 | 95.5 (88.9-98.8) | 62.9 (60.6-65.1) | 11.2 (9.1-13.7) | 99.6 (99.1-99.9) | 0.79 |
| *Wb123* Ab or *Bm33* Ab | 89 | 1142 | 51.87 | <0.001 | 96.6 (90.5-99.3) | 41.7 (39.4-44.0) | 7.5 (6.1-9.2) | 99.6 (98.8-99.9) | 0.69 |
| *Bm14* Ab or *Bm33* Ab | 89 | 1067 | 65.53 | <0.001 | 97.8 (92.1-99.7) | 45.9 (43.5-48.2) | 8.2 (6.6-10) | 99.8 (99.1-100) | 0.72 |
| Any Ab | 89 | 1162 | 52.56 | <0.001 | 97.8 (92.1-99.7) | 40.6 (38.3-42.9) | 7.5 (6.0-9.2) | 97.8 (92.1-99.7) | 0.69 |
| *Wb123* Ab and *Bm14* Ab | 89 | 352 | 314.82 | <0.001 | 89.9 (81.7-95.3) | 85.0 (83.2-86.6) | 22.7 (18.5-27.5) | 99.4 (98.9-99.7) | 0.87 |
| *Wb123* Ab and *Bm33* Ab | 89 | 570 | 177.79 | <0.001 | 93.3 (85.9-97.5) | 73.1 (71.0-75.1) | 14.6 (11.8-17.7) | 99.5 (99.0-99.8) | 0.83 |
| *Bm14* Ab and *Bm33* Ab | 89 | 408 | 250.56 | <0.001 | 88.8 (80.3-94.5) | 81.8 (80-83.6) | 19.4 (15.6-23.5) | 99.3 (98.8-99.7) | 0.85 |
| All Ab | 89 | 344 | 314.2 | <0.001 | 88.8 (80.3-94.5) | 85.4 (83.6-87) | 23.0 (18.6-27.8) | 99.4 (98.8-99.7) | 0.87 |

*Blue: 0-50.0%; green: 50.1-75.0%; yellow: 75.1-90.0%; orange: >90%. Ab = antibody, Ag = Antigen, NPV = negative predictive value, PPV = positive predictive value, ROC = area under the Receiver-Operating Characteristic Curve.*
