# Supplementary material for: Sensitivity of anti-filarial antibodies for lymphatic filariasis surveillance: Insights from a serological survey in Samoa in 2018
Source: PLoS Negl Trop Dis. 2025 Jan 30;19(1):e0012835. doi: 10.1371/journal.pntd.0012835 (PMC11922241; doi:10.1371/journal.pntd.0012835)
Supplement: S6 Table — Light blue denotes low relative risk ratio (RRR), dark green denotes high RRR. (DOCX) [file pntd.0012835.s006.docx]

**Supplementary Table 6: Unadjusted and adjusted multinomial logistic regression analysis for testing positive to different antigen (Ag) and antibody (Ab) combinations by age group and sampling design in relation to participants aged 5-9 years old in randomly selected primary sampling units (PSUs), Samoa 2018.** Colour spectrum from blue to yellow to green indicates progressive increase in risk. Light blue denotes low relative risk ratio (RRR), dark green denotes high RRR.

| **Age group**  **(Sampling design)** | **5-9 years* (Random PSU)** | **≥10 years**  **(Random PSU)** | | | | | **5-9 years**  **(Purposive PSU)** | | | | | **≥10 years**  **(Purposive PSU)** | | | | |
| --- | --- | --- | --- | --- | --- | --- | --- | --- | --- | --- | --- | --- | --- | --- | --- | --- |
|  | **N (%)** | **N (%)** | **RRR**  **(95% CI)** | ***P*-value** | **aRRR**  **(95% CI)** | **P-value** | **N (%)** | **RRR**  **(95% CI)** | ***P*-value** | **aRRR**  **(95% CI)** | **P-value** | **N (%)** | **RRR**  **(95% CI)** | ***P*-value** | **aRRR**  **(95% CI)** | **P-value** |
| **Total** | **1,641** | **1,636** |  |  |  |  | **255** |  |  |  |  | **263** |  |  |  |  |
| Ag-positive | 24 (1.3) | 62 (4.1) | 3.3 (2.1, 5.1) | <0.001 | 3.4 (2.1, 5.3) | <0.001 | 4 (2.1) | 1.6 (0.7, 4.0) | 0.278 | 1.6 (0.7, 4.1) | 0.252 | 27 (11.4) | 9.5 (4.8, 18.7) | <0.001 | 10.2 (5.1, 20.2) | <0.001 |
| *Bm14* Ab | 123 (6.8) | 335 (22.2) | 3.8 (2.7, 5.2) | <0.001 | 4.0 (2.8, 5.6) | <0.001 | 24 (9.4) | 1.4 (0.6, 3.2) | 0.385 | 1.4 (0.6, 3.3) | 0.387 | 101 (37.8) | 7.8 (4.7, 12.9) | <0.001 | 8.5 (5.1, 14.2) | <0.001 |
| *Bm33* Ab | 509 (30.3) | 853 (54.3) | 2.7 (2.0, 3.5) | <0.001 | 2.7 (2.1, 3.6) | <0.001 | 111 (41.3) | 1.6 (0.9, 2.8) | 0.085 | 1.6 (0.9, 2.8) | 0.082 | 186 (67.2) | 4.6 (2.7, 7.9) | <0.001 | 4.7 (2.8, 8.1) | <0.001 |
| *Wb123* Ab | 265 (15.6) | 538 (34.4) | 2.8 (2.3, 3.3) | <0.001 | 2.9 (2.4, 3.4) | <0.001 | 49 (18.5) | 1.2 (0.8, 1.8) | 0.311 | 1.2 (0.8, 1.8) | 0.306 | 135 (49.7) | 5.0 (3.1, 8.2) | <0.001 | 5.4 (3.3, 8.9) | <0.001 |
| *Wb123* Ab or *Bm14* Ab | 313 (18.4) | 609 (38.9) | 2.8 (2.3, 3.3) | <0.001 | 2.9 (2.3, 3.5) | <0.001 | 58 (21.5) | 1.2 (0.8, 1.8) | 0.359 | 1.2 (0.8, 1.9) | 0.357 | 148 (54.0) | 4.9 (3.1, 7.7) | <0.001 | 5.3 (3.3, 8.3) | <0.001 |
| *Wb123* Ab or *Bm33* Ab | 592 (35.2) | 947 (60.2) | 2.7 (2.1, 3.6) | <0.001 | 2.8 (2.1, 3.7) | <0.001 | 119 (44.4) | 1.5 (0.9, 2.4) | 0.124 | 1.5 (0.9, 2.4) | 0.118 | 195 (71.3) | 4.4 (2.5, 7.7) | <0.001 | 4.6 (2.6, 8.0) | <0.001 |
| *Bm14* Ab or *Bm33* Ab | 527 (31.6) | 878 (56.0) | 2.7 (2.0, 3.6) | <0.001 | 2.8 (2.1, 3.7) | <0.001 | 878 (56.0) | 1.6 (1.0, 2.6) | 0.072 | 1.6 (1.0, 2.6) | 0.069 | 189 (67.9) | 4.5 (2.6, 7.8) | <0.001 | 4.6 (2.7, 8.0) | <0.001 |
| Ag or *Bm14* Ab | 126 (7.0) | 339 (22.3) | 3.7 (2.7, 5.2) | <0.001 | 3.9 (2.8, 5.6) | <0.001 | 26 (10.4) | 1.6 (0.7, 3.5) | 0.286 | 1.6 (0.7, 3.7) | 0.291 | 105 (40.4) | 8.5 (5.0, 14.3) | <0.001 | 9.3 (5.5, 15.6) | <0.001 |
| Ag or *Bm33* Ab | 510 (30.4) | 856 (54.4) | 2.7 (2.0, 3.6) | <0.001 | 2.7 (2.1, 3.6) | <0.001 | 112 (42.0) | 1.7 (0.9, 2.9) | 0.068 | 1.7 (1.0, 2.9) | 0.072 | 187 (68.1) | 4.7 (2.7, 8.2) | <0.001 | 4.9 (2.8, 8.4) | <0.001 |
| Ag or *Wb123* Ab | 270 (15.8) | 541 (34.5) | 2.7 (2.3, 3.3) | <0.001 | 2.8 (2.4, 3.4) | <0.001 | 51 (19.4) | 1.3 (0.8, 1.9) | 0.238 | 1.3 (0.8, 2.0) | 0.237 | 137 (51.1) | 5.2 (3.2, 8.4) | <0.001 | 5.6 (3.5, 9.2) | <0.001 |
| Ag or *Bm14* Ab or *Bm33* Ab | 527 (31.6) | 879 (56.1) | 2.7 (2.0, 3.6) | <0.001 | 2.8 (2.1, 3.7) | <0.001 | 114 (42.8) | 1.6 (1.0, 2.7) | 0.059 | 1.6 (1.0, 2.7) | 0.057 | 190 (68.8) | 4.6 (2.6, 8.1) | <0.001 | 4.8 (2.7, 8.3) | <0.001 |
| Ag or *Bm14* Ab or *Wb123* Ab | 315 (18.4) | 611 (39.0) | 2.8 (2.3, 3.3) | <0.001 | 2.9 (2.3, 3.5) | <0.001 | 60 (22.5) | 1.3 (0.8, 2.0) | 0.273 | 1.3 (0.8, 2.0) | 0.274 | 150 (55.4) | 5.2 (3.3, 8.1) | <0.001 | 5.6 (3.6, 8.7) | <0.001 |
| Ag or *Wb123* Ab or *Bm33* Ab | 593 (35.3) | 949 (60.3) | 2.7 (2.1, 3.6) | <0.001 | 2.8 (2.1, 3.7) | <0.001 | 120 (45.1) | 1.5 (0.9, 2.5) | 0.106 | 1.5 (0.9, 2.5) | 0.101 | 196 (72.1) | 4.6 (2.6, 8.1) | <0.001 | 4.7 (2.7, 8.4) | <0.001 |
| *Wb123* Ab or *Bm14* Ab or *Bm33* Ab | 608 (36.4) | 964 (61.2) | 2.7 (2.1, 3.5) | <0.001 | 2.8 (2.1, 3.6) | <0.001 | 119 (44.4) | 1.4 (0.9, 2.3) | 0.181 | 1.4 (0.9, 2.3) | 0.173 | 198 (72.0) | 4.3 (2.5, 7.7) | <0.001 | 4.5 (2.6, 7.9) | <0.001 |
| LF-seropositive | 608 (36.4) | 965 (61.3) | 2.7 (2.1, 3.5) | <0.001 | 2.8 (2.1, 3.6) | <0.001 | 120 (45.1) | 1.4 (0.9, 2.4) | 0.151 | 1.4 (0.9, 2.4) | 0.144 | 199 (72.8) | 4.5 (2.5, 8.1) | <0.001 | 4.7 (2.6, 8.4) | <0.001 |

**Reference group; aRRR = adjusted relative risk ratio (adjusted for sex).*
