# Supplementary material for: Sensitivity of anti-filarial antibodies for lymphatic filariasis surveillance: Insights from a serological survey in Samoa in 2018
Source: PLoS Negl Trop Dis. 2025 Jan 30;19(1):e0012835. doi: 10.1371/journal.pntd.0012835 (PMC11922241; doi:10.1371/journal.pntd.0012835)
Supplement: S7 Table — (DOCX) [file pntd.0012835.s007.docx]

**Supplementary Table 7: Unadjusted and adjusted multinomial logistic regression analysis for testing positive to different antigen (Ag) and antibody (Ab) combinations among participants aged ≥10 years old and primary sampling unit (PSU) selection, Samoa 2018.**

|  | **≥10 years old**  **(Randomly selected PSU)*** | **≥10 years old**  **(Purposively selected PSU)** | | | | |
| --- | --- | --- | --- | --- | --- | --- |
|  | **N (%)** | **N (%)** | **RRR**  **(95% CI)** | ***P*-value** | **aRRR**  **(95% CI)** | ***P*-value** |
| **Total** | **1636** | **263** |  |  |  |  |
| Ag-positive | 62 (4.1) | 27 (11.4) | 2.9 (1.5, 5.5) | **0.002** | 3.0 (1.6, 5.7) | **0.001** |
| *Bm14* Ab | 335 (22.2) | 101 (37.8) | 2.1 (1.3, 3.3) | **0.003** | 2.2 (1.4, 3.4) | **0.002** |
| *Bm33* Ab | 853 (54.3) | 186 (67.2) | 1.7 (1.0, 2.9) | 0.052 | 1.7 (1.0, 3.0) | **0.047** |
| *Wb123* Ab | 538 (34.4) | 135 (49.7) | 1.8 (1.1, 2.9) | **0.014** | 1.9 (1.2, 3.1) | **0.011** |
| *Wb123* Ab or *Bm14* Ab | 609 (38.9) | 148 (54.0) | 1.8 (1.1, 2.8) | **0.017** | 1.8 (1.1, 3.0) | **0.013** |
| *Wb123* Ab or *Bm33* Ab | 947 (60.2) | 195 (71.3) | 1.6 (0.9, 2.9) | 0.108 | 1.6 (0.9, 3.0) | 0.099 |
| *Bm14* Ab or *Bm33* Ab | 878 (56.0) | 189 (68.0) | 1.6 (0.9, 2.9) | 0.080 | 1.7 (0.9, 2.9) | 0.074 |
| Ag or *Bm14* Ab | 339 (22.3) | 105 (40.4) | 2.3 (1.4, 3.7) | **0.002** | 2.4 (1.5, 3.9) | **<0.001** |
| Ag or *Bm33* Ab | 856 (54.4) | 187 (68.1) | 1.8 (1.0, 3.0) | **0.046** | 1.8 (1.0, 3.1) | **0.042** |
| Ag or *Wb123* Ab | 541 (34.5) | 137 (51.1) | 1.9 (1.2, 3.1) | **0.008** | 2.0 (1.2, 3.2) | **0.006** |
| Ag or *Bm14* Ab or *Bm33* Ab | 879 (56.1) | 190 (68.8) | 1.7 (1.0, 3.0) | 0.069 | 1.7 (1.0, 3.1) | 0.064 |
| Ag or *Bm14* Ab or *Wb123* Ab | 611 (39.0) | 150 (55.4) | 1.9 (1.2, 3.0) | **0.009** | 1.9 (1.2, 3.1) | **0.007** |
| Ag or *Wb123* Ab or *Bm33* Ab | 949 (60.3) | 196 (72.1) | 1.7 (0.9, 3.0) | 0.098 | 1.7 (0.9, 3.1) | 0.089 |
| *Wb123* Ab or *Bm14* Ab or *Bm33* Ab | 964 (61.2) | 198 (72.0) | 1.6 (0.9, 2.9) | 0.120 | 1.6 (0.9, 3.0) | 0.111 |
| LF-seropositive | 965 (61.3) | 199 (72.9) | 1.7 (0.9, 3.1) | 0.107 | 1.7 (0.9, 3.1) | 0.098 |

**Reference value; aRRR = adjusted relative risk ratio (adjusted for sex).*
