# Supplementary figures and images for: Sensitivity of anti-filarial antibodies for lymphatic filariasis surveillance: Insights from a serological survey in Samoa in 2018
Source: PLoS Negl Trop Dis. 2025 Jan 30;19(1):e0012835. doi: 10.1371/journal.pntd.0012835 (PMC11922241; doi:10.1371/journal.pntd.0012835)

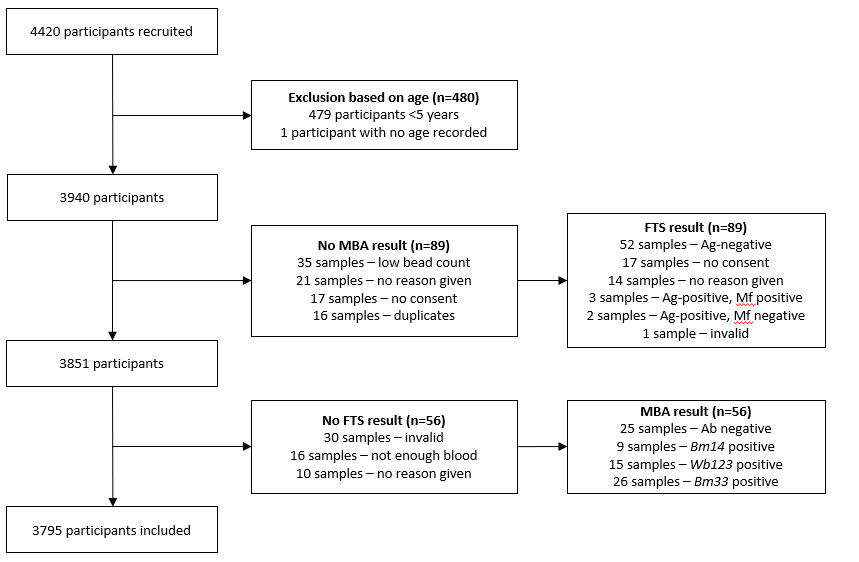


**Supplementary Fig 1: Flow chart of participant inclusion and exclusion, Samoa 2018**

Supplement: S1 Fig — (DOCX) [file pntd.0012835.s008.docx]
