# Supplementary material for: Sensitivity of anti-filarial antibodies for lymphatic filariasis surveillance: Insights from a serological survey in Samoa in 2018
Source: PLoS Negl Trop Dis. 2025 Jan 30;19(1):e0012835. doi: 10.1371/journal.pntd.0012835 (PMC11922241; doi:10.1371/journal.pntd.0012835)
Supplement: S2 Fig — (DOCX) [file pntd.0012835.s009.docx]

|  | **Participants ≥5 years** | **Participants 5-9 years** | **Participants ≥10 years** |
| --- | --- | --- | --- |
| ***Bm14* Ab vs Ag** |   *rho*=0.754, *p*-value<0.001 |   *rho*=0.470, *p*-value=0.004 |   *rho*=0.806, *p*-value<0.001 |
| ***Wb123* Ab vs Ag** |   *rho*=0.660, *p*-value<0.001 |   *rho*=0.579, *p*-value<0.001 |   *rho*=0.723, *p*-value<0.001 |
| ***Bm33* Ab vs Ag** |   *rho*=0.613, *p*-value<0.001 |   *rho*=0.589, *p*-value<0.001 |   *rho*=0.655, *p*-value<0.001 |
|  | | | |

**Supplementary Fig 2: Pearson’s correlation coefficient estimates for the relationship between antigen (Ag) prevalence and antibody (Ab) prevalence in participants aged ≥5 years, 5-9 years and ≥10 years at the primary sampling unit (PSU) level, Samoa 2018**
