# Supplementary material for: Sensitivity of anti-filarial antibodies for lymphatic filariasis surveillance: Insights from a serological survey in Samoa in 2018
Source: PLoS Negl Trop Dis. 2025 Jan 30;19(1):e0012835. doi: 10.1371/journal.pntd.0012835 (PMC11922241; doi:10.1371/journal.pntd.0012835)
Supplement: S3 Fig — (DOCX) [file pntd.0012835.s010.docx]

|  | **Participants ≥5 years** | **Participants 5-9 years** | **Participants ≥10 years** |
| --- | --- | --- | --- |
| ***Bm14* Ab vs *Bm33* Ab** |   *rho*=0.875, *p*-value<0.001 |   *rho*=0.707, *p*-value<0.001 |   *rho*=0.884, *p*-value<0.001 |
| ***Bm14* Ab vs *Wb123* Ab** |   *rho*=0.912, *p*-value<0.001 |   *rho*=0.753, *p*-value<0.001 |   *rho*=0.935, *p*-value<0.001 |
| ***Bm33* Ab vs *Wb123* Ab** | ****  *rho*=0.900, *p*-value<0.001 | ****  *rho*=0.782, *p*-value<0.001 | ****  *rho*=0.875, *p*-value<0.001 |
|  | | | |

**Supplementary Fig 3: Pearson’s correlation coefficient estimates for the relationship between antibody (Ab) prevalence in participants aged ≥5 years, 5-9 years and ≥10 years at the primary sampling unit (PSU) level, Samoa 2018**
