# Supplementary material for: Sensitivity of anti-filarial antibodies for lymphatic filariasis surveillance: Insights from a serological survey in Samoa in 2018
Source: PLoS Negl Trop Dis. 2025 Jan 30;19(1):e0012835. doi: 10.1371/journal.pntd.0012835 (PMC11922241; doi:10.1371/journal.pntd.0012835)
Supplement: S4 Fig — (DOCX) [file pntd.0012835.s011.docx]

**Supplementary Fig 4: Sensitivity, specificity, and area under the Receiver-Operating Characteristic Curve (ROC) of antigen (Ag), antibodies (Abs), and combinations of Abs for 5-9-year-olds (TOP) and ≥10-year-olds (BOTTOM), Samoa 2018.**
