# Supplementary material for: Sensitivity of anti-filarial antibodies for lymphatic filariasis surveillance: Insights from a serological survey in Samoa in 2018
Source: PLoS Negl Trop Dis. 2025 Jan 30;19(1):e0012835. doi: 10.1371/journal.pntd.0012835 (PMC11922241; doi:10.1371/journal.pntd.0012835)
Supplement: S5 Fig — Vales below 1.0 indicate a reduced risk of seropositivity. (DOCX) [file pntd.0012835.s012.docx]

**Supplementary Fig 5: Adjusted relative risk ratio (RRR) of testing positive to antigen (Ag), antibody (Ab), and a combination of markers, among participant’s aged ≥10 years old from purposively selected primary sampling units (PSUs), using participants ≥10 years old from randomly selected PSUs as the referent, Samoa 2018.** Values above 1.0 indicate and increased risk of seropositivity for the Ag, Ab, or combination of Ag and Ab listed on the Y-axis. Vales below 1.0 indicate a reduced risk of seropositivity.
